# Co-treatment with therapeutic neural stem cells expressing carboxyl esterase and CPT-11 inhibit growth of primary and metastatic lung cancers in mice

**DOI:** 10.18632/oncotarget.2547

**Published:** 2014-10-24

**Authors:** Bo-Rim Yi, Seung U. Kim, Kyung-Chul Choi

**Affiliations:** ^1^ Laboratory of Biochemistry and Immunology, College of Veterinary Medicine, Chungbuk National University, Cheongju, Chungbuk, Republic of Korea; ^2^ Department of Medicine, Faculty of Medicine, University of British Columbia, Vancouver, British Columbia, Canada

**Keywords:** Lung cancer, metastasis, interferon-beta, 5-fluorocytosine, stem cell therapy

## Abstract

In this study, neural stem cells (NSCs)-derived enzyme/prodrug therapy (NDEPT) was used to treat primary lung cancer or metastatic lung cancer in the brain. To confirm the anti-tumor effect of NSCs expressing carboxyl esterase (CE), A549 lung cancer cells were treated with HB1.F3.CE cells and CPT-11. A significant decrease in the viability/proliferation of lung cancer cells was observed compared to negative controls or cells treated with CPT-11 alone. To produce a mouse model of primary lung cancer or lung cancer metastasis to the brain, A549 cells were implanted in the dorsal area of the mouse or right hemisphere. CM-DiI pre-stained stem cells were implanted near the primary lung cancer tumor mass or in the contralateral brain. Two days after stem cells injection, mice were inoculated with CPT-11 (13.5 kg/mouse/day) via intraperitoneal injection. In the primary lung cancer mouse models, tumor mass was 80% lower in response to HB1.F3.CE in conjunction with CPT-11, while it was only reduced by 40% in the group treated with CPT-11 alone. Additionally, therapeutic efficacy of co-treatment with stem cells and CPT-11 was confirmed by detection of apoptosis and necrosis in primary and metastatic lung cancer tissues. By secreting VEGF, tumor cells modulate Erk1/2 and Akt signaling and migration of stem cells. This further increased tumor-selectivity of stem cell/prodrug co-therapy. Overall, these results indicate that NSCs expressing the therapeutic gene may be a powerful tool for treatment of primary lung cancer or metastasis of lung cancer to the brain.

## INTRODUCTION

Neural stem cells (NSCs) that have the capacity to self-renew and differentiate into neurons and glia can be grown from adult subventricular zones (SVZ) [1]. In the adult brain, NSCs exist in two regions, the SVZ of the lateral ventricle, and the subgranular zone of the hippocampal dentate gyrus. Neurogenesis occurs continuously in these regions [2]. NSCs have been shown to migrate to areas of injury site as well as brain pathological areas, such as ischemic and neoplastic lesions [3]. Therefore, migration of endogenous and exogenous NSCs to areas of pathology is critical to tissue regeneration. Directed cell migration is initiated in response to various cytokines and growth factors, and receptors such as stromal cell derived factor (SDF-1α)/CXCR4, stem cell factor (SCF)/c-Kit, and vascular endothelial growth factor (VEGF)/VEGFR have been shown to affect stem cell migration [4]. In a previous study, hepatocyte growth factor (HGF) activity on mouse mesenchymal stem cells (MSCs) isolated from bone marrow was investigated in terms of proliferation, migration and cell differentiation [5]. Additionally, several signal pathways such as phosphatidylinositol 3-kinase (PI3K)/Akt signaling regulate survival, proliferation, differentiation, and migration of stem cells [6]. Akt regulates proliferation of embryonic mouse NSCs and neuronal differentiation by affecting the cell cycle regulators cyclin D, cyclin-dependent kinase inhibitor p27Kip1, and p21Cip1/Waf1 [7]. Increased Akt activity via elevated phosphorylation of PI3K promotes stem cells migration *in vitro* and *in vivo*, as well as proliferation and apoptotic signaling [8]. Inhibition of PI3K by LY294002, a selective PI3K inhibitor, decreases neural progenitor cell proliferation and migration [9]. Moreover, MSCs demonstrate an increased migratory propensity in the presence of basic fibroblast growth factor (bFGF) through a PI3K/Akt pathway [10].

VEGF is a critical mediator of angiogenesis and tumor proliferation that is frequently overexpressed in a variety of cancers [11]. In another study, transplantation of VEGF-expressing NSCs provided neuroprotection against hemorrhage and improved functional recovery in intracerebral hemorrhage (ICH) animal models [12]. An investigation of VEGF and platelet-derived growth factor alpha/beta (PDGFαβ) also showed that VEGF could stimulate migration through the PDGF receptor, confirming the intricacies involved in the signaling induction [13]. In another study, inhibition of neurogenesis in the dentate gyrus of adult mice by blocking VEGFR2 significantly impaired animal learning capability [14].

NSCs-based therapies for Parkinson's disease, Huntington's disease, multiple sclerosis, spinal cord injury and primary and metastatic cancer metastasis to the brain have been successfully developed based on these principles [15]. The efficacy of modalities employing NSCs-directed enzyme/prodrug therapy (NDEPT) has been examined in various animal models of human primary and metastatic cancers [16]. Enzyme/prodrug therapy can be designed to selectively target tumor cells over normal cells, which can minimize side-effects [17]. Several types of enzymes employed in enzyme/prodrug therapy are capable to converting non-toxic prodrugs into toxic agents, including cytosine deaminase (CD) and carboxyl esterase (CE) [18]. The CE gene is most widely used to promote formation of SN-38 from irinotecan (CPT-11) [19]. The active form, SN-38, is a strong mammalian topoisomerase I inhibitor that is 1,000-fold more potent then CPT-11 and induces the accumulation of double strand DNA breaks in dividing cancer cells [20]. In a previous study, treatment with CPT-11 not only decreased the number of HB1.F3.CE cells, but also inhibited the growth of untransfected neighboring ovarian cancer cells [21].

In this study, we employed an immortalized HB1.F3 and HB1.F3.CE expressing CE gene for treatment of primary and metastatic lung cancer. Lung cancer is the second most frequent type of tumor and a major problem in human health [22]. Of all malignancies, primary lung cancer has the highest incidence of brain metastasis, and approximately 40% of all patients with lung cancer develop such metastasis [23]. Metastatic lung cancer is a debilitating disease that results in a high burden of symptoms and poor quality of life with an estimated prognosis after diagnosis of less than one year [24, 25]. The goal of the current study was to examine the effects of NSCs expressing therapeutic genes with prodrug treatment on primary and metastatic lung cancer to the brain. Thus, therapeutic efficacy of HB1.F3.CE in the presence of a prodrug, CPT-11, was investigated in the primary and metastatic mouse models by diverse molecular methods. In addition to therapeutic effects, the migratory ability of HB1.F3.CE was observed by a transwell migration assay *in vitro* and *in vivo*. We also elucidated the mechanism of migratory properties related to VEGF/VEGFR2 signaling by regulating the expression of the downstream proteins, Erk1/2 and Akt. Overall, the results of this study suggested that NDEPT using cells expressing the CE gene may be an excellent therapeutic system for selective targeting of metastatic lung cancer to the brain, as well as primary lung cancer.

## RESULTS

### Antitumor effect of HB1.F3.CE and CPT-11 in A549 lung cancer cells

**Figure 1:**
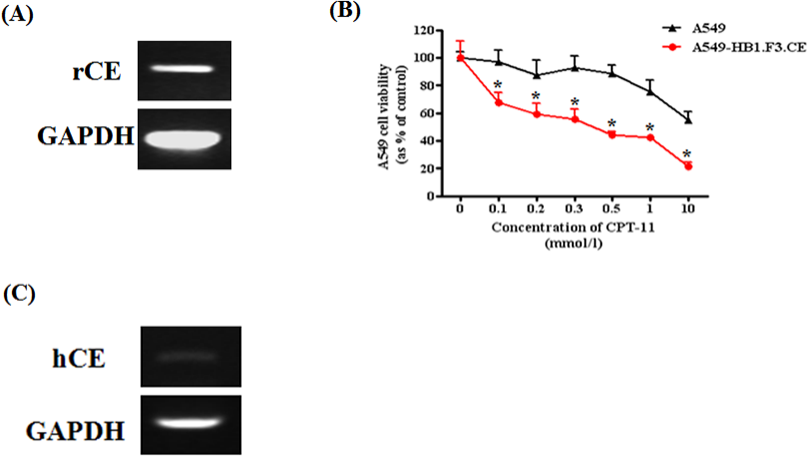
Expression of therapeutic gene in HB1.F3.CE cells and anticancer effect of stem cells with prodrug against A549 lung cancer cells Total RNA was extracted from cancer and stem cells, and PCR was conducted to amplify the human and rabbit CE gene, respectively. PCR product was separated by 1.5% agarose gel electrophoresis. GAPDH was used as a positive. **(A)** Expression of rabbit CE gene in stem cells. **(B)** Effect of HB1.F3.CE and CPT-11 co-treatment. CPT-11 and stem cells were co-treated in 96-well plates following cancer cell seeding. In the control well, stem cells were not co-cultured in the plate. Four days after prodrug treatment, MTT assay was conducted to measure cell viability. **(C)** Expression of human CE gene in A549 lung cancer cells. Each experiment was conducted in triplicate and results are presented as the mean ± SD. *; p < 0.05 *vs.* CPT-11 treated cells without HB1.F3.CE cells.

Therapeutic NSCs expressing the CE gene, HB1.F3.CE cells, were used to investigate whether growth of A549 lung cancer cells is reduced in response to co-treatment with stem cells and the prodrug, CPT-11. To accomplish this, we tested the expression of rabbit CE gene in stem cells by RT-PCR. HB1.F3.CE cells expressed the exogenous CE gene at 237 bp (Fig. 1A). Cell viability of A549 lung cancer cells was measured by MTT assay following co-culture with CPT-11 at 0.1, 0.2, 0.3, 0.5, 1.0, or 10.0 μg/ml. In the CPT-11 only treated group, low levels of CPT-11 (0.1 μg/ml) did not alter the proliferation of A549 lung cancer cells, whereas growth of stem cells and prodrug co-treated cells was reduced by 35% under the same conditions (Fig. 1B). Furthermore, the proliferation rate of cancer cells was significantly inhibited by 60% in the co-treated group relative to the CPT-11 single treated group, in which proliferation was reduced by approximately 40% in response to high concentrations of CPT-11 (1.0 μg/ml). Moreover, to explain the cytotoxic of CPT-11 on cancer cells in the absence of the stem cells, we identified the endogenous human CE gene in A549 lung cancer cells at 182 bp (Fig. 1C).

### Inhibition of tumor growth by HB1.F3.CE and CPT-11

**Figure 2:**
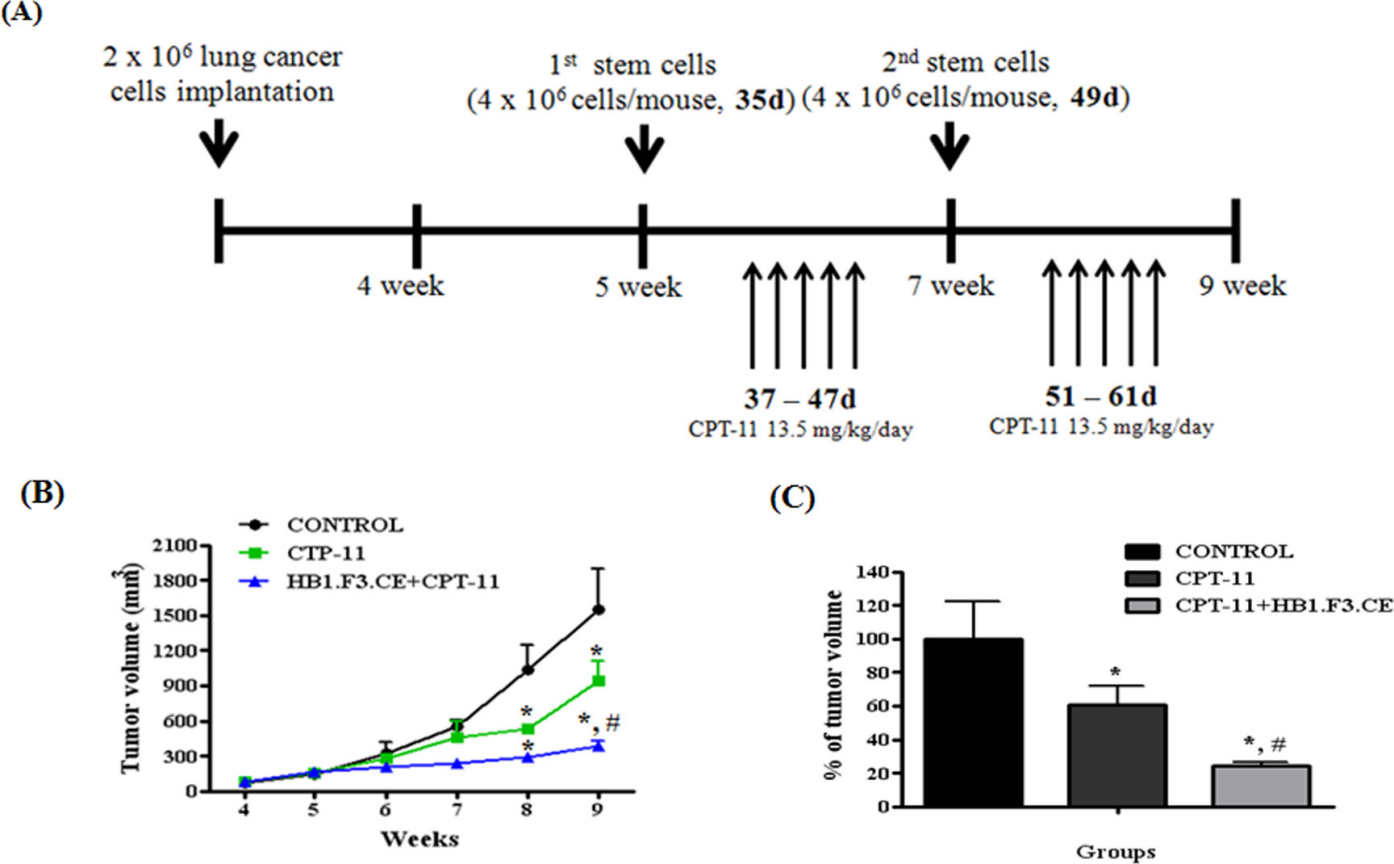
Schedule of stem cell therapy in the primary lung cancer model **(A)** Scheme of treatment. Primary lung cancer models were produced by implanting A549 cells (2 × 10^6^ cells/mouse) subcutaneously in the dorsal region of male BALB/c nude mice. Five weeks after inoculation with cancer cells, CM-DiI stem cells (4 × 10^6^ cells/mouse) were injected near formed tumor masses and CPT-11 (13.5 mg/kg/day) was administered via intraperitoneal injection (*i.p.*). **(B)** Alteration of tumor mass volume. Tumor volume was measured every week and calculated by 0.5236 × length × width × height. **(C)** The relative volume of tumor mass at week nine. Each experiment was represented as the mean ± SEM. *; p < 0.05 *vs.* negative control (no treatment with stem cells or CPT-11). #; p < 0.05 *vs.* CPT-11 treated cells without HB1.F3.CE cells.

To determine whether HB1.F3.CE inhibited the growth of primary lung cancer mass, an animal study using BALB/c nude mice was performed with stem cells and CPT-11. As shown in Figure 2A, A549 lung cancer cells were injected into the dorsal area of the mouse and then co-treated with stem cells/CPT-11 during the experimental period. Lung cancer tumor burden was reduced in the CPT-11+/−HB1.F3.CE treated groups relative to the negative control group (Fig. 2B). Differences in tumor mass were observed at three weeks following treatments with the stem cells and a prodrug. Following treatment for four weeks, tumor volume was significantly reduced by 80% in the HB1.F3.CE with CPT-11 co-treated group, whereas it was only reduced by 40% in CPT-11 treated mice (Fig. 2C).

### Histopathological analysis of tumor mass excised from mice

**Figure 3:**
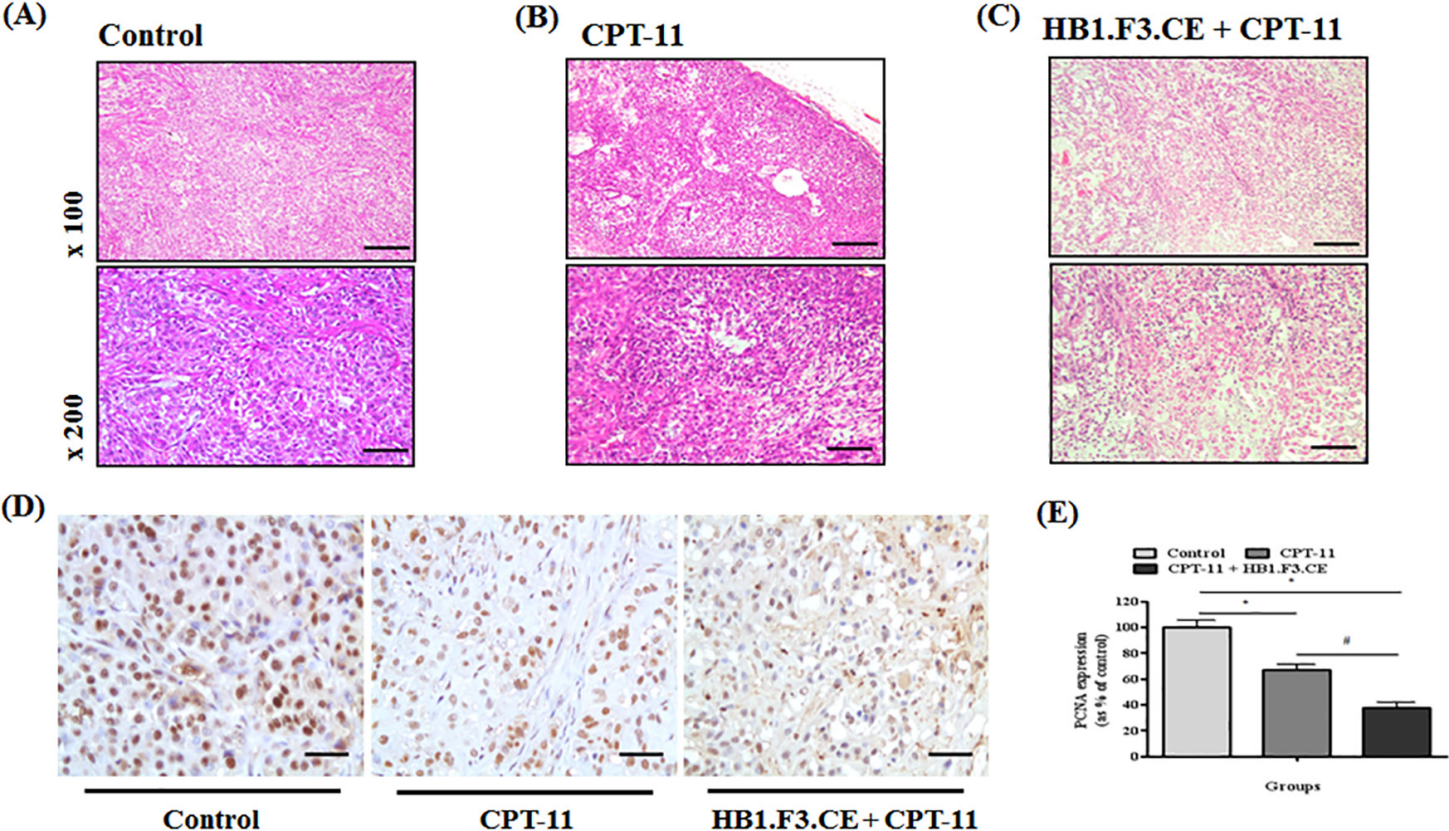
Immunohistochemical (IHC) staining and hematoxylin and eosin (H&E) staining After sacrifice of experimental mice, tumors were excised and fixed in 10% normal formalin. Samples were then stained with H&E to confirm histopathological analysis and features of necrosis or apoptosis. Additionally, PCNA protein as a proliferation marker was detected in tissue specimens using a mouse monoclonal anti-PCNA primary antibody (1:100 dilution). Following incubation with primary antibody, biotinylated anti-mouse secondary antibody was applied to the slide (1:500 dilution). **(A)** Tumor mass of negative control (H&E staining). **(B)** Tumor mass of CPT-11 treated mice (H&E staining). **(C)** Tumor mass of HB1.F3.CE cells and CPT-11 co-treated mice (H&E staining). **(D)** IHC staining for PCNA protein in each tumor mass. **(E)** The relative value of PCNA protein expression levels. *; p < 0.05 *vs.* negative control (no treatment with stem cells or CPT-11). #; p < 0.05 *vs.* CPT-11 treated cells without HB1.F3.CE cells. Magnification × 100 or × 200.

To further analyze the effects of stem cells expressing therapeutic genes, H&E staining of tumor mass obtained from the mice was conducted. In the negative control, features of tumor cells such as aggressive tendency, high density, and large nuclear inner cancer cells were observed (Fig. 3A). Conversely, apoptosis or necrosis of lung cancer cells was observed in the CPT-11+/−HB1.F3.CE cell treated mice (Fig. 3B). Additionally, features of necrosis and apoptosis such as nuclear pyknosis, karyorrhexis, and karyolysis occurred more frequently in HB1.F3.CE cells and CPT-11 co-treated mice relative to those treated with CPT-11 alone (Fig. 3C). Moreover, the density of tumor cells was significantly reduced in the co-treated group owing to dissolution of the nucleus.

We also investigated expression of the proliferation marker, PCNA protein, by IHC staining of tissue specimens. Proliferative tumor cells displayed strong nuclear staining in the negative control group relative to the CPT-11+/−HB1.F3.CE cell treated group (Fig. 3D). Specifically, the relative value of PCNA expression was significantly decreased by 70% in the tumor burden of HB1.F3.CE and CPT-11 treated mice (Fig. 3E).

### Tumor tropism of stem cells in primary lung cancer mouse models

**Figure 4:**
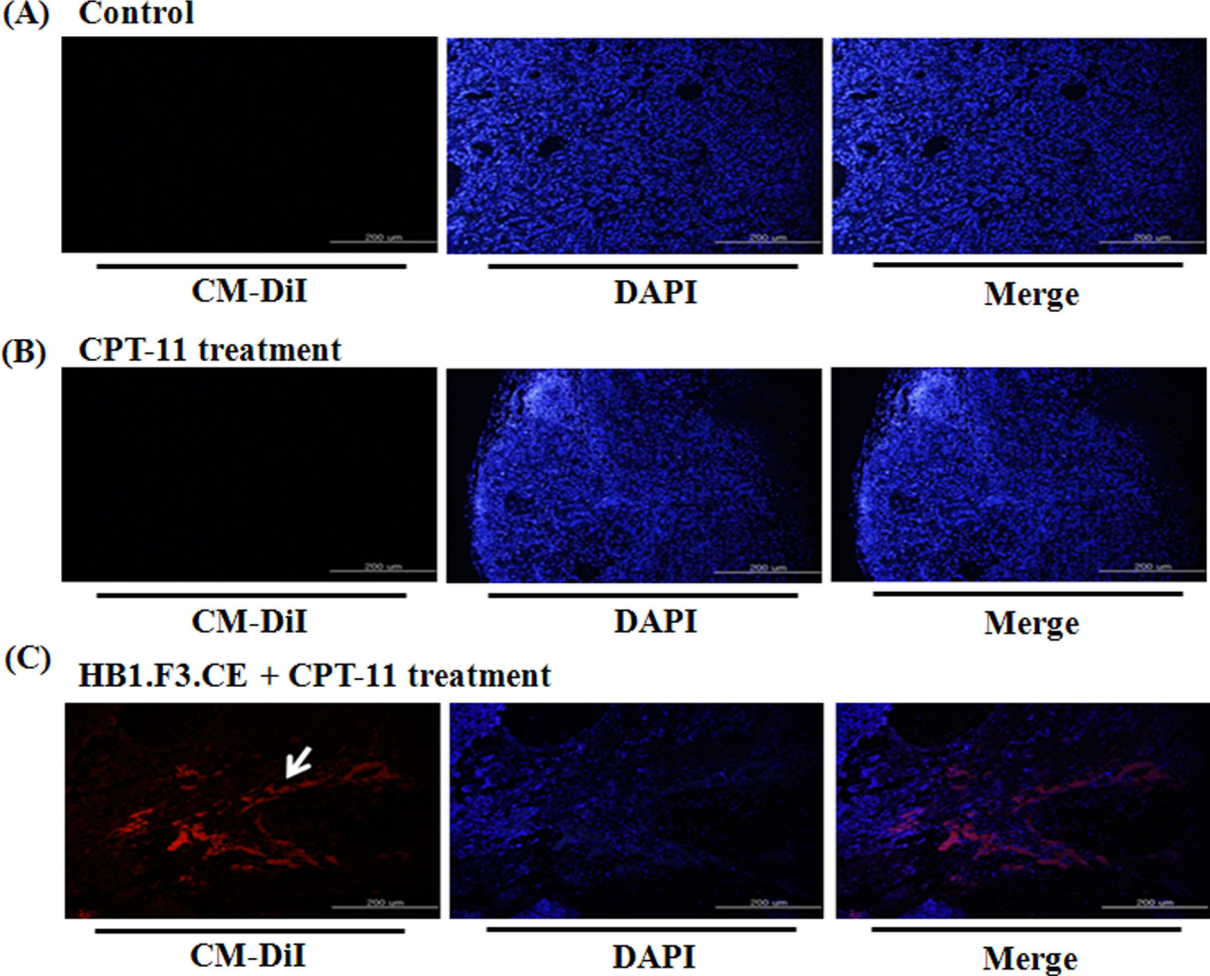
The migratory ability of human neural stem cells in tumor burden The migratory ability of human NSCs toward A549 lung cancer cells was determined using animal models. **(A)** Negative control. **(B)** CPT-11 treated mice. **(C)** HB1.F3.CE cells and CPT-11 co-treated mice. Red fluorescence: CM-DiI stained cytoplasm of HB1.F3.CE cells. Blue fluorescence: DAPI stained nucleus of A549 lung cancer cells and HB1.F3.CE cells. White arrow: stained stem cells. Magnification × 100.

We next tested whether the stem cells migrated toward lung cancer burden using a fluorescence analysis. Before injection of stem cells into the mice, HB1.F3.CE cells were stained with red fluorescent CM-DiI. No red fluorescence labeled stem cells were detected in the negative control and CPT-11 single treated groups (Figs. 4A and B). However, red fluorescence was observed in the tumor burden of the HB1.F3.CE and CPT-11 treated group (Fig. 4C).

### Effect of stem cells on lung cancer metastasis to the brain in mouse models

**Figure 5:**
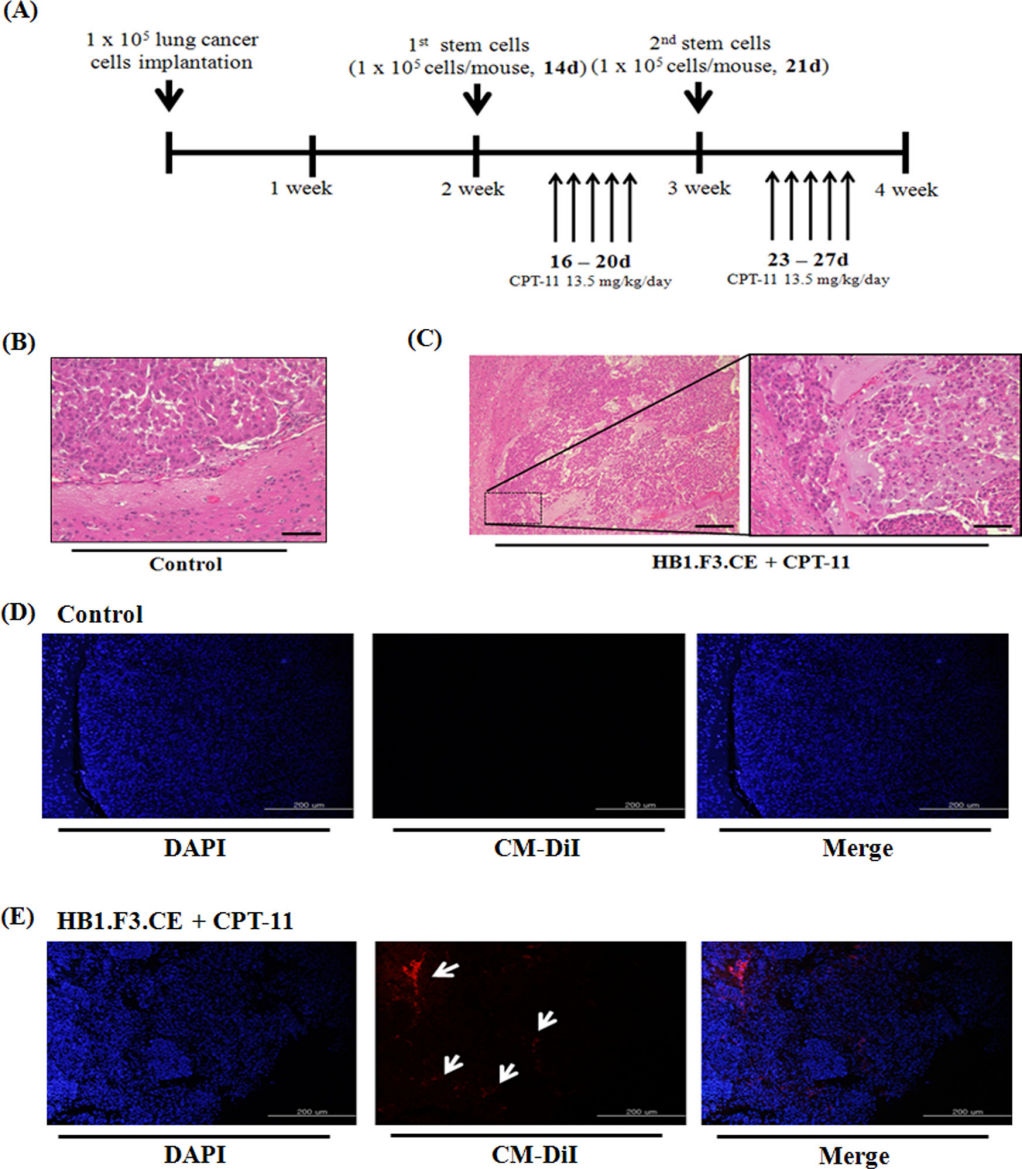
Histopathological analysis and tumor-tropic effect of stem cells in metastatic lung cancer animal models A549 lung cancer cells (1 × 10^5^ cells/mouse) were implanted in the right hemisphere of mice and CM-DiI pre-stained stem cells (1 × 10^5^ cells/mouse) were injected into the left hemisphere two times. To induce the therapeutic effect of stem cells, CPT-11 (13.5 mg/kg/day) was administered by intraperitoneal injection (*i.p.*) for five days. After final prodrug injection, all of the mouse brain was excised and the specimen was subjected to hematoxylin and eosin (H&E) staining for histological analysis. **(A)** Scheme of metastatic lung cancer animal models. **(B)** Negative control brain specimen. **(C)** HB1.F3.CE and CPT-11 co-treated brain specimen. **(D)** Negative control group. The *in vivo* migratory ability was determined by fluorescence analysis through DAPI staining of brain specimens. **(E)** HB1.F3.CE and CPT-11 treated group. Blue fluorescence: DAPI stained nucleus of A549 lung cancer cells and HB1.F3.CE cells. White arrow: migrated stem cells. Magnification × 100 and × 200.

To evaluate the therapeutic effects of stem cells and prodrug, we produced metastatic lung cancer mouse models by injecting cancer cells directly into the right hemisphere of the brain (Fig. 5A). At 2 weeks after inoculation, red fluorescence labeled HB1.F3.CE cells were injected into the left hemisphere and prodrug was administered via the abdominal cavity. The antitumor effect of CPT-11 and HB1.F3.CE cells was evaluated at 4 weeks after initial lung cancer cells were injected. Upon histological analysis, tumor burden was detected in the all of mouse brain and differences were observed between normal brain tissues and lung cancer cells (Fig. 5B). Alternatively, liquefactive necrosis was identified in HB1.F3.CE treated mice in the presence of CPT-11 (Fig. 5C). Additionally, cancer cells underwent pyknosis and karyorrhexis.

### Migratory effect of stem cells in metastatic lung cancer mouse model

In addition to primary lung cancer mouse models, we inoculated cancer cells into the brain of animal models following CM-DiI fluorescence staining of HB1.F3.CE cells. To investigate the migration properties, cancer cells were injected into the right hemisphere and pre-stained HB1.F3.CE cells were injected into the opposite site of the brain. No red fluorescence was detected near the tumor burden in the right hemisphere of the mouse brains in the control (Fig. 5D), while red spots were observed in the HB1.F3.CE plus CPT-11 co-treated group by fluorescent microscopy (Fig. 5E).

### Role of VEGF/VEGFR2 signaling

**Figure 6:**
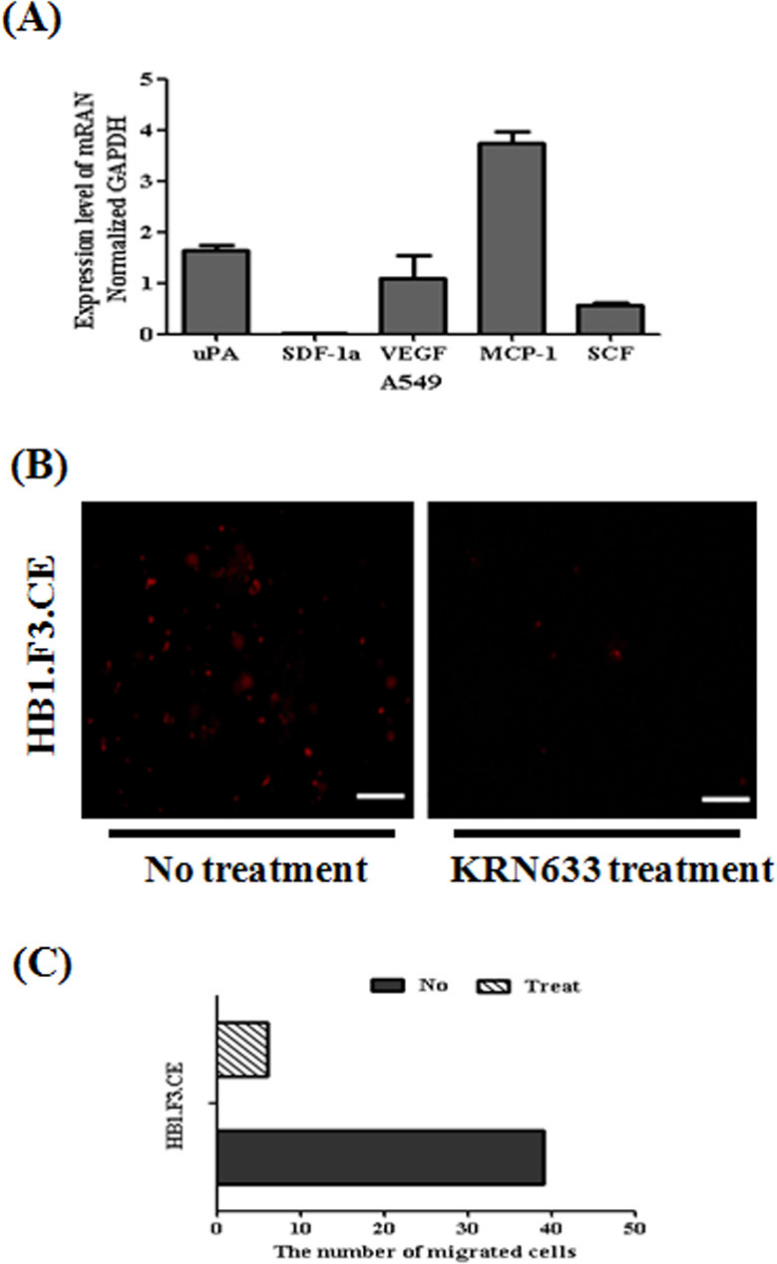
Assessment of tumor tropic effect through vascular endothelial growth factor and their receptor 2 (VEGF/VEGFR2) **(A)** Several chemoattractant factors secreted by cancer cells. To confirm the migration-related factor, total RNA was obtained from A549 lung cancer cells. Real time PCR for chemoattractant factors including uPA, SDF-1α, VEGF, MCP-1, and SCF secreted by cancer cells was then conducted. **(B)** Inhibition of migratory effect following treatment with the VEGFR2 inhibitor, KRN633. To induce inhibition of the interaction of VEGR and VEGFR2, stem cells were treated with 100 μM KRN633 before *in vitro* migration assay. One hour after KRN633 treatment, CM-DiI stained stem cells were seeded in lung cancer pre-cultured plates. Migrated stem cells were detected by microscopy after one day. **(C)** Bar diagram to demonstrate the amount of migrated stem cells after KRN633 treatment. Red fluorescence: CM-DiI stained cytoplasm of HB1.F3.CE cells. Magnification × 200.

To explain the migratory ability of stem cells against tumor cells, several chemoattractant factors including uPA, SDF-1α, VEGF, MCP-1, and SCF were examined and quantified by real time PCR in A549 lung cancer cells (Fig. 6A). In this study, A549 lung cancer cells mainly secreted uPA, VEGF, MCP-1, and SCF. We also confirmed that VEGF/VEGFR2 signaling induced the migration of HB1.F3.CE cells toward A549 lung cancer cells by a VEGFR2 inhibition assay. Before transwell assay of cancer and stem cells, stem cells were pre-stained with CM-DiI and pre-treated with 100 μM KRN633 for one hour to inhibit the interaction between VEGF and VEGFR2 secreted by cells. After culture of stem cells in the upper chamber of the transwell for one day, we confirmed that the amount of migrated stem cells against lung cancer cells was significantly decreased relative to stem cells not treated with KRN633 (Fig. 6B). Approximately eight cells migrated in one transwell of non-treated cells, which was about 75% lower than that of KRN633 treated cells (Fig. 6C).

### Mechanism of inhibition of migratory effects via Erk1/2 and Akt

**Figure 7:**
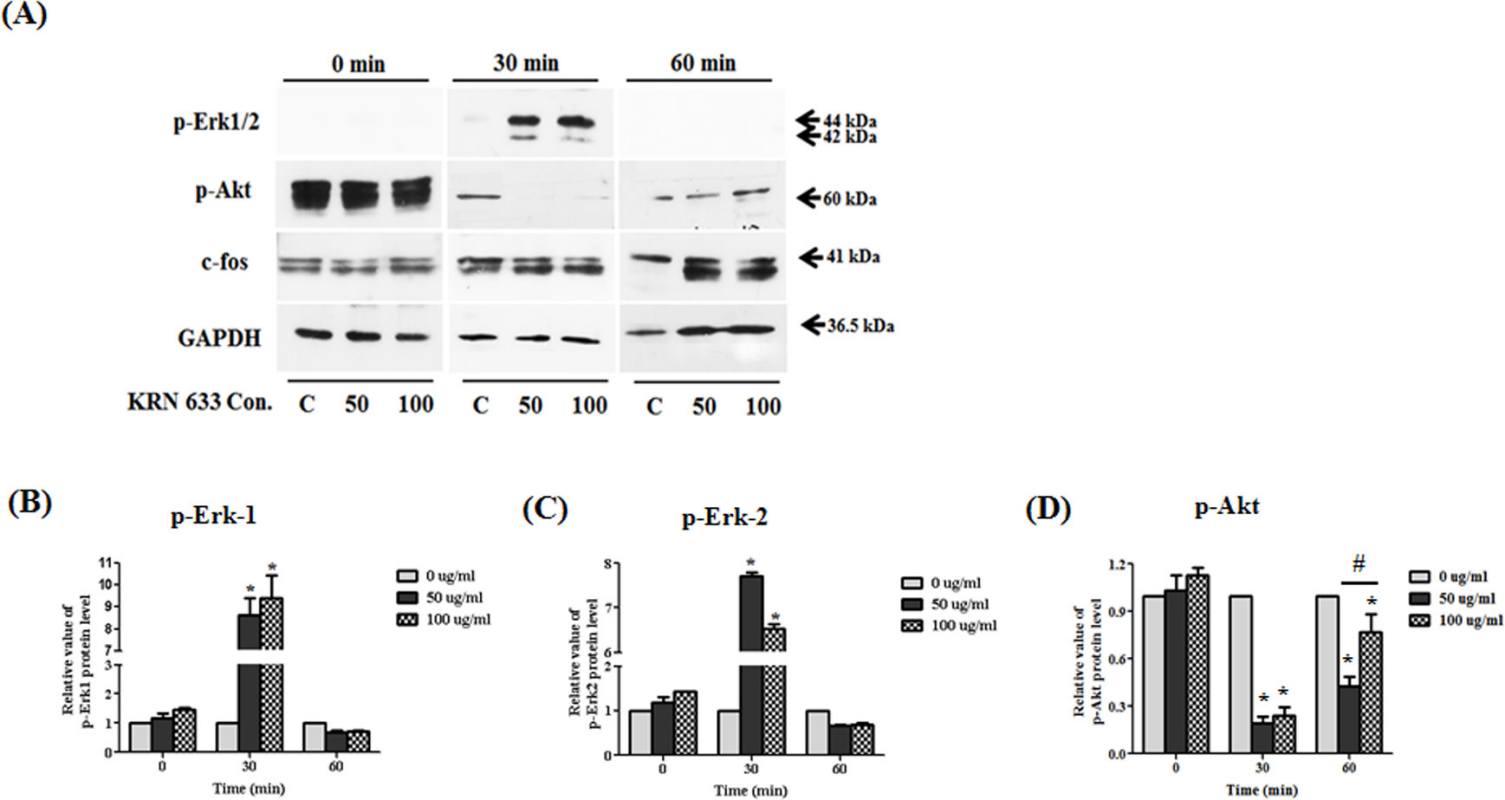
Alteration of downstream regulated migration ability Whole lysates of KRN633 treated HB1.F3.CE cells were extracted using protein extraction solution. Quantified proteins were then separated by SDS-PAGE and transferred to a PVDF membrane. For immunoblotting, the membrane was incubated with primary antibodies including anti-phospho-Erk1/2 (1:1,000 dilution), anti-phospho-Akt (1:1,000 dilution), and anti-c-fos (1:2,000 dilution). Each protein was normalized against the GAPDH protein. **(A)** Expression of p-Erk1/2, p-Akt, and c-fos in KRN633 treated HB1.F3.CE cells. **(B)** The relative value of p-Erk1 protein. **(C)** The relative value of p-Erk2 protein. **(D)** The relative value of p-Akt protein. Each experiment was conducted in triplicate and presented as the mean ± SD. *; p < 0.05 *vs.* negative control (no treatment with CPT-11 or HB1.F3.CE cells). #; p < 0.05 *vs.* CPT-11 treated cells without HB1.F3.CE cells.

To determine the effects of VEGF/VEGFR2 signaling in stem cells, we inhibited VEGFR2 using a KRN633. To accomplish this, 50 or 100 μM KRN633 was applied to pre-cultured stem cells for 0, 30, and 60 min before protein extraction. To confirm the alteration of VEGF/VEGFR2 signaling via KRN633 treatment, we assessed several downstream proteins related to stimulation of VEGF/VEGFR2 signaling (Fig. 7A). We observed a dramatic increase in p-Erk1/2 protein in response to treatment with 50 or 100 μM KRN633 (Figs. 7B and C). Conversely, selective downregulation of endogenous VEGFR2 led to decreased Akt phosphorylation in stem cells within one hour of inhibitor treatment (Fig. 7D). The rate of Akt phosphorylation was increased at 60 min after treatment of stem cells with KRN633. The relative value of c-fos protein did not change significantly in response to KRN633 treatment (data not shown).

## DISCUSSION

The development of an effective and safe therapy for primary or metastatic cancers is one of the major challenges in the treatment of cancer [26]. In this study, we focused on the antitumor effects of therapeutic NSCs, which are known to have the ability to convert prodrugs to the active form. A cell viability assay confirmed the effects of the CE gene expressed in HB1.F3.CE cells on A549 lung cancer cells. Specifically, cancer cell viability decreased significantly by 80%, whereas viability of cancer cells treated with CPT-11 alone was reduced to approximately 40%. CPT-11 is classified as a non-toxic prodrug and a carbamate that is hydrolyzed by CEs to yield SN-38 [27]. Typically, less than 5% of the drug is converted into the active form, SN-38, when CPT-11 is administered to humans [28]. In mice, more than 50% of the CPT-11 is hydrolyzed to SN-38 within the first hour of treatment [29]. The reason for this discrepancy may be differences in the levels of CE gene expression among species or the proficiency of drug hydrolysis of the different CE genes. We selected the rabbit CE gene for NDPET because structural similarity was observed between rabbit CE and human CE (81% amino-acid identity) [30]. In this study, reduction of cell viability in CTP-11 single treated cells may have occurred owing to the weak expression of the CE gene. This study also demonstrated the effects of HB1.F3.CE plus CPT-11 on the mouse model for primary lung cancer and lung cancer metastasis to the brain. In the primary lung cancer models, therapeutic stem cells dramatically inhibited tumor growth by up to 80% in the presence of CPT-11, while CPT-11 alone led to a 40% reduction.

Upon histopathological analysis, we frequently observed apoptosis and necrosis-related features, as well as a reduction of tumor cell density in the tissue specimen of primary and metastatic lung cancer animal models upon H&E staining. In the second approach, we used IHC staining to detect proliferative cells in tissue specimens. A nearly 2.5-fold reduction in the number of PCNA-positive cells per section was observed in the HB1.F3.CE and CPT-11 co-treatment tissue. We also used IHC staining to compare inhibition of tumor burden in response to treatment with CPT-11 alone and observed a decrease of up to 40% when compared with negative control mice. Together, these two sets of histological data indicated a dramatic reduction in cancer mass during stem cell therapy in the presence of CPT-11. Similar to the *in vitro* results, the therapeutic effects of CPT-11 may be induced by endogenous CE gene *in vivo* assay.

Moreover, the migratory effect of HB1.F3.CE cells was shown in primary and metastatic lung cancer animal models upon fluorescence analysis. DM-DiI pre-stained HB1.F3.CE cells were inoculated into areas near the tumor burden of primary cancer models or the opposite hemisphere of the brain in the metastatic cancer model and red-fluorescence was detected in each tumor mass. The tumor tropic effect of NSCs expressing therapeutic genes has been investigated in various xenograft mouse models and cancer cells, such as pancreatic, cervical and gastric cancers, as well as brain tumors [31–33]. To evaluate the mechanism of the migratory effects, we investigated whether several chemoattractant factors induced the migration of stem cells secreted by A549 lung cancer cells. In a previous study, specific migration of MSCs was reportedly guided by chemokines [34]. Moreover, other researchers reported that signaling by SDF-1α and their receptor, CXCR4, is important to regulation of the migration of different types of stem/progenitor cells [35]. In this study, real-time PCR analysis demonstrated that A549 lung cancer cells expressed several factors, including uPA, VEGF, MCP-1, and SCF. Yi *et al.* and others reported that some receptors were mainly expressed in HB1.F3 cells, including uPA-related receptor (uPAR), VEGFR2, and SCF-related receptor (c-Kit) [19, 36]. Therefore, we investigated whether the migratory effects of stem cells toward cancer cells were mediated through VEGF/VEGFR2 signaling due to induction by various cancer cells. VEGF is an angiogenic factor known to be a primary factor involved in angiogenesis [37]. VEGF-targeted therapies were initially developed with the notion that they would inhibit new blood vessels in patients with advanced-stage malignancies [38]. In the present study, VEGF/VEGFR2 signaling was inhibited by pretreatment of HB1.F3.CE cells with 100 μM KRN633 for one hour before *in vitro* migration assay. Evaluation of migrated HB1.F3.CE cells by fluorescence microscopy revealed that the number of stem cells was significantly decreased by 75% in inhibitor treated stem cells.

Furthermore, alteration of VEGF/VEGFR2-related downstream proteins was investigated following KRN633 treatment. Interestingly, we found that phosphorylation of Erk1/2 was induced and phosphorylation of Akt was decreased in stem cells via inhibition of VEGF/VEGFR2 signaling at early time. In the previous study, neural progenitor cells (NPCs) differentiation and migration was paralleled by changes in the phosphorylation of Erk1/2, which is involved in a wide range of functions including regulation of neurogenesis [39]. Additionally, Shinjyo *et al.* reported that SDF-1α/CXCR4 signaling stimulated NPCs migration through Erk signaling [40]. In addition to migration, Erk1/2 played an important role in regulation of the differentiation and proliferation of NSCs, which was mediated by Cdk2, cyclin D, and Hes1 protein [41]. Therefore, these findings suggest that increased p-Erk1/2 appears to be related with the differentiation status rather than migration ability of stem cells after VEGF/VEGFR2 signaling inhibition. In the case of Akt protein, brain-derived neurotrophic factor (BDNF) increased the phosphorylation of Akt, a downstream target of the PI3K pathway; however, this enhanced effect was abolished when neural stem/progenitor cells were pre-treated with the PI3K inhibitor, LY294002 [42]. In this study, we found that Akt was dephosphorylated at early time and phosphorylated at later time following the treatment with a VEGFR2 inhibitor in the stem cells, suggesting that the migratory ability of NSCs may be inhibited via Akt dephosphorylation at early time and activation of Erk1/2 in MAPK pathways may induce Akt re-phosphorylation to influence the differentiation of stem cells at later time. Taken together, the migratory effects of stem cells were regulated by the downstream VEGF/VEGFR2 signaling factors, PI3K/Akt and Erk1/2, in this study.

In summary, our *in vitro* and *in vivo* data indicate that stem cells expressing the therapeutic gene CE, HB1.F3.CE cells, display therapeutic effects towards primary and metastatic lung cancer to the brain. In the presence of low levels of CPT-11, SN-38 may be efficiently converted by the CE gene with minimal side effects. Additionally, the mechanism of the migratory ability of stem cells suggests that VEGF/VEGFR2 signaling, one of several chemoattractant factors secreted by the cancer cells, may play an important role in migration through regulation of Erk1/2 and Akt phosphorylation. In conclusion, stem cells expressing a therapeutic gene have therapeutic potential for treatment of metastatic cancer and primary cancer as an efficient delivery vehicle for therapeutic genes.

## MATERIALS AND METHODS

### Cell culture

The lung cancer cell line, A549, was obtained from the Korea cell line bank (KCLB, Seoul, Korea) and grown in Dulbecco's modified eagle's medium (DMEM; Hyclone Laboratories, Inc., Logan, UT, USA) supplemented with 10% (v/v) heat inactivated fetal bovine serum (FBS; Hyclone Laboratory Inc.), antibiotic agent consisting of 100 Unit/ml penicillin and 100 μg/ml streptomycin (Cellgro Mediatech Inc., Manassas, VA, USA) and 10 mM HEPES (Gibco, Carlsbad, CA, USA). Immortalized human NSCs, HB1.F3.CE cells, were provided by Chungang University (Seoul, Korea) and incubated in DMEM supplemented with 10% FBS, 100 Unit/ml penicillin and 100 μg/ml streptomycin, 10 mM HEPES and 0.1% antimycoplasmal agents (Invivogen, San Diego, CA, USA). All cell lines were incubated at 37°C in a humidified 5% CO_2_ atmosphere and subcultured using 0.05% trypsin/0.02% EDTA (Gibco).

### Therapeutic effects of HB1.F3.CE and CPT-11 against lung cancer cells

Lung cancer cell viability was measured by an MTT assay according to the manufacturer's protocols. Briefly, A549 lung cancer cells (1650 cells/well/100 μl media) were placed in a 96-well plate, and then incubated overnight at 37°C. The next day, HB1.F3.CE (3350 cells/well/100 μl media) or vehicle (medium) was added to the existing cancer cells in the 96-well plate. We then applied different concentrations of CPT-11 (Sigma-Aldrich Co. St. Louis, MO, USA) as a prodrug (0.1, 0.2, 0.3, 0.5, 1.0, and 10.0 mmol/l). After four days, 10 μl of 3-(4-,5-dimethylthiazol-2-yl)-2,5-dyphenyl tetrazolium bromide (MTT; Sigma-Aldrich Co.) reagent was added to each well, and insoluble formazan crystal was dissolved in dimethyl sulfoxide (DMSO; Junsei Chemical, Tokyo, Japan), then incubated at 37°C for four hours. The absorbance of reduced MTT was measured at 540 nm using a VERSA man microplate reader (Molecular Devices, Sunnyvale, CA, USA). The percent cell viability was plotted using GraphPad Prism (v5.0; GraphPad Software, San Diego, CA, USA). Each data point indicates the average of triplicate measurements (n = 12).

### Semi-quantitative reverse transcription PCR and quantitative real-time PCR

Total RNA was isolated from A549 lung cancer cells using TRIzol RNA extraction solution (Invitrogen Lift Technologies, Carlsbad, CA, USA). Briefly, 1 μg of RNA from HB1.F3.CE and A549 lung cancer cells was reverse transcribed with murine leukemia virus reverse transcriptase (MMLV-RT; iNtRON Biotechnology, Sungnam, Kyeonggido, Korea), 10 pM dNTP (Bioneer, Deajeon, Korea), 200 pM monomer random primer (TaKaRa Bio., Shiga, Japan), 5 × RT buffer (iNtRON Biotechnology) and RNase inhibitor (iNtRON Biotechnology). cDNA was then synthesized at 37°C for one hour, after which the reaction was stopped by incubation at 95°C for 5 min.

The cDNA produced from the extracted total RNA of HB1.F3.CE and A549 lung cancer cells was amplified by PCR to confirm the expression of human or rabbit CE genes. The reaction mixture contained 2.5 Units Taq polymerase (iNtRON Biotechnology), 5 pM dNTP, 10 × PCR buffer (iNtRON Biotechnology), and 10 pM of each primer set (Table 1). PCR was conducted by subjecting the samples to the following conditions: denaturation at 95°C for 30 s, annealing at 58°C for 30 s, and extension at 72°C for 30 s for 30 cycles. The RT-PCR products were resolved on 1.5% agarose gels by electrophoresis, stained with ethidium bromide (EtBr, Sigma-Aldrich Co.), visualized on a UV transilluminator, and photographed digitally using the Gel Doc 2000 apparatus (BioRad Laboratories, Hercules, CA, USA). Expression of glyceraldehyde 3-phosphate dehydrogenase (GAPDH) was used as a loading control.

**Table 1:** Primers used in semi-RT PCR and real time PCR

mRNA		Sequence (5′->3′)
rCE	Forward	TGCTGGGCTATCCACTCTCT
	Reverse	CTCCAGCATCTCTGTGGTGA
hCE	Forward	CACTCCTGCTGACTTGACCA
	Reverse	CATCC CCTGTGCTGAAGAAT
uPA	Forward	GGCAGGCAGATGGTCTGTAT
	Reverse	TTGCTCACCACAACGACATT
SDF-1α	Forward	GTGTCACTGGCGACACGTAG
	Reverse	TCCCATCCCACAGAGAGAAG
MCP-1	Forward	CAAGCAGAAGTGGGTTCAGGA
	Reverse	TCTTCGGAGTTTGGGTTTGC
VEGF	Forward	CCAGCACATAGGAGAGATGAGCTT
	Reverse	TCTTTCTTTGGTCTGCATTCACAT
SCF	Forward	GGCAAATCTTCCAAAAGACTACA
	Reverse	GCCTTCAGAAATATTTGAAAACTTG
GAPDH	Forward	ATGTTCGTCATGGGTGTGAACCA
	Reverse	TGGCAGGTTTTTCTAGACGGCAG

To quantify the expression of chemoattractant factors such as urokinase-type plasminogen activator (uPA), SDF-1α, VEGF, monocyte chemotactic protein 1 (MCP-1), and SCF in A549 lung cancer cells, cDNA was quantified by real time PCR, which was conducted using a 2 × SYBR green premix (TaKaRa Bio.), ROX dye (TaKaRa Bio.), and reverse and forward primers and the following reaction conditions (Table 1): denaturation at 95°C for 15 s, annealing at 58°C for 20 s, and extension at 72°C for 15 s (40 cycles). All data were analyzed by the comparative 2^−ΔΔCt^ method and normalized relative to the GAPDH gene.

### Primary lung cancer mouse models

In this study, six-week-old male BALB/c nude mice were purchased from Central Laboratory Animal (Seoul, Korea). All protocols involving animals were reviewed and approved by the Animal Care Committee of Chungbuk National University and performed in accordance with the guide for the Care and Use of Experimental Animals. The mice were cared for under a pathogen free environment with a 12 h light/dark cycle and frequent ventilation. Additionally, all mice were freely fed autoclaved rodent diet (Central Lab. Animal Inc.) and distilled water. A549 lung cancer cells (2 × 10^6^ cells/100 μL/mouse) suspended in phosphate buffered saline (PBS) were subcutaneously injected with matrigel (BD Biosciences, Franklin Lakes, NJ, USA) into the dorsal area. When the volume of the tumor burden reached 200 mm^3^, the 24 mice were randomly divided into three groups: 1) HB1.F3.CE and CPT-11 treated group, 2) CPT-11 treated group, and 3) negative control (untreated with stem cells or prodrug). On week 5 post-inoculation, an HB1.F3.CE suspension containing 4 × 10^6^ cells was injected two times into the nearby lung cancer burden (at day 35 and 49). Prior to injection, stem cells pre-stained with 2 μM chloromethylbenzamide-1, 1′-dioctadecyl-3,3,3′-tetramethyl-indocarbocyanine perchlorate (CM-DiI; Invitrogen Life Technologies). A prodrug, CPT-11 (Sigma-Aldrich Co.), was injected into the mice with HB1.F3.CE cells for 11 days after stem cell injection. It was given via an intraperitoneal injection at a dose 13.5 mg/kg/day in 100 μL of saline. Tumor sizes were measured with a caliper and calculated every week using the following formula: length × width × height × 0.5236. The mice were sacrificed 48 h after the last treatment and the tumor masses were then excised for histopathological analysis.

### Xenograft model of lung cancer metastasis to brain

A total of 21 six-week old male nude mice were purchased from Central Laboratory Animal. A549 lung cancer cells (1 × 10^5^ cells/8 μl/mouse) were directly implanted into the white matter of the right hemisphere [anterior/posterior (AP) +1.0 mm, medial/lateral (ML) +1.7 mm, dorsal/ventral (DV) −3.2 mm] to produce metastatic lung cancer models. Two weeks later, 21 mice were randomly divided into three groups: 1) HB1.F3.CE and CPT-11 treated group, 2) CPT-11 treated group, and 3) a negative control (untreated with stem cells or prodrug). Mice in the HB1.F3.CE and CPT-11 treated group had 2 μM CM-DiI pre-stained HB1.F3.CE at 1 × 10^5^ cells/mice injected into the left hemisphere of the brain. CPT-11 (13.5 mg/kg/day; Sigma-Aldrich Co.) was then administered intraperitoneally (*i.p.*) once a day, 5 days per week for 2 weeks.

### Hematoxylin and eosin (H&E) staining and fluorescence analysis

The tumor burdens and brains were collected after sacrificing the mice, fixed in 4% normal formalin (Sigma-Aldrich Co.) and embedded in paraffin blocks. Next, 3 μm sections were cut using a sliding microtome, after which the tumor burdens and brains were stained with hematoxylin and eosin (Sigma-Aldrich Co.). The sections were then examined for apoptosis, necrosis and other histological aspects. Additionally, the fluorescence of the migrated stem cells in the tumor burden and brain were evaluated by DAPI (4′, 6-diamidino-2-phenylindole; Sigma-Aldrich Co.) staining at 200 ng/ml. Stem cells were pre-stained with 2 μM CM-DiI (Invitrogen Life Technologies) staining solution and injected into primary and metastatic lung cancer animal models. DAPI staining was applied as a counterstain on the slide. All stained slides were observed by light microscopy using a BX51 microscope (Olympus, Tokyo, Japan).

### Immunohistochemistry (IHC)

IHC of the specimen slides prepared from primary lung cancer mice was conducted. To expose the antigen in tissues, antigen retrieval was conducted by placing samples in 0.01 M citrate buffer (pH 6.0) and microwaving for 10 min. Next, samples were incubated with 0.3% methanol/hydrogen peroxidase (Sigma-Aldrich Co.) for 30 min at room temperature to quench the endogenous peroxidase. Slides were then blocked using 5% bovine albumin serum (BAS, Sigma-Aldrich Co.) supplemented 10% normal goat serum (Vector Laboratories, Burlingame, CA, USA) for 1 h at room temperature, then washed twice in 1 × phosphate buffered saline (PBS) containing 0.05% Tween 20. Samples were subsequently incubated with a primary mouse monoclonal antibody against proliferating cell nuclear antigen (PCNA, 1:100, Abcam, plc., Cambridge, UK) for overnight at 4°C. After four washes for 10 min in PBS-Tween, the slides were incubated in the biotinylated anti-mouse secondary antibody (1:500 dilution, Vector Laboratories) solution for 30 min at 37°C. To form the immunoreactive complex, ABC kit reagent (Vectastain Universal Elite ABC kit, Vector Laboratories) was treated at each slide for 30 min. After DAB substrate (Sigma-Aldrich Co.) and hematoxylin staining solution treatment, all slides were detected under a BX51 light microscope for digital photography.

### Transwell assay

Transwell migration was performed to determine the migration abilities of HB1.F3.CE cells. Briefly, A549 lung cancer cells were seeded in 24-well plates and incubated at 37°C for one day. After fibronectin (concentration 250 μg/ml, Sigma-Aldrich Co.) pre-coating of the bottom of a transwell (8 μM pore, BD Biosciences), CM-DiI pre-stained stem cells were seeded in the upper chamber of the transwell and incubated for one day. Dose response comparison of the effects of 100 μM of the VEGFR2 inhibitor, KRN633 (Selleckchem, Houston, TX, USA), on migratory properties of HB1.F3.CE stem cells was conducted before seeding the transwells. Inhibitor was applied to the stem cells culture dish for one hour, detached from the culture plate through trypsin/EDTA treatment, and then cultured in the upper chamber of transwell. Following stem cells incubation, non-migrated cells were removed from the transwell using a cotton swab and migrated stem cells were fixed in cold methanol for ten min. Red fluorescent stem cells was then detected by fluorescence microscopy (IX71 inverted microscope; Olympus, Tokyo, Japan).

### Immunoblotting

HB1.F3.CE cells were plated in DMEM medium containing 10% FBS at densities of 1.0 × 10^5^ cells/dish in 60 mm^3^ dishes and incubated overnight at 37°C. The following day, cells were exposed to 50 or 100 μM KRN633 in medium for one hour. Dishes were then washed twice with 1 × phosphate buffered saline (PBS) within one hour, after which 1 × RIPA protein extraction solution (mixed with 50 mM Tris-HCl, pH 8.0, 150 mM NaCl, 1% NP-40, 0.5% deoxycholic acid, and 0.1% sodium dodecyl sulfate) was added. Extracted proteins were subsequently quantified by bicinchoninic acid (BCA; Sigma-Aldrich Co.) and copper (II) sulfate (Sigma-Aldrich Co.), after which 40 μg of protein was size fractionated by 12% sodium dodecyl sulfate-polyacrylamide gel electrophoresis (SDS-PAGE) and transferred onto polyvinylidene difluoride (PVDF) transfer membrane (BioRad Laboratories Inc.). Membranes were blocked with 5% non-fat dry milk in Tris-buffered saline-0.1% Tween 20 (TBS-T, BioRad Laboratories Inc.) and probed with anti-phospho-Erk1/2 (1:1,000 dilution, Cell signaling Technology, Inc., Danvers, MA, USA ), anti-phospho-Akt (1:1,000 dilution, Cell Signaling Technology, Inc.), anti-c-fos (1:2,000 dilution, Abcam plc.), and anti- GAPDH (1:1,000 dilution, Santa Cruz Biotechnology, Inc. CA, USA) primary IgG antibodies. After overnight incubation with primary antibodies, membranes were incubated with horseradish peroxidase linked goat anti-rabbit (1:3000 dilution, Santa Cruz Biotechnology, Inc.) or anti-mouse (1:3000 dilution, BioRad Laboratories Inc.) secondary IgG antibody for 2 h. The proteins were then detected using an enhanced chemiluminescence (ECL) kit (West-Q Chemiluminescent Substrate Plus Kit, GenDEPOT, Barker, TX, USA). Intensity values were determined with the Quantity One analysis program (BioRad Laboratories), and the percentage of protein was normalized against cells that were not treated with KRN633. Finally, data were plotted using the GraphPad prism software package.

### Statistical analysis

Results obtained from specific experiments were analyzed using the GraphPad Prism software and data were expressed as the mean ± SD. or SEM in cellular or mouse models and compared by one-way ANOVA, followed by Tukey's test to compare all of the groups against a control. A P < 0.05 was considered significant.
